# The Production of Human β-Glucocerebrosidase in *Nicotiana benthamiana* Root Culture

**DOI:** 10.3390/ijms19071972

**Published:** 2018-07-06

**Authors:** Uthailak Naphatsamon, Takao Ohashi, Ryo Misaki, Kazuhito Fujiyama

**Affiliations:** International Center for Biotechnology, Osaka University, Suita-shi, Osaka 565-0871, Japan; uthailak@icb.osaka-u.ac.jp (U.N.); ohashi@icb.osaka-u.ac.jp (T.O.); misaki@icb.osaka-u.ac.jp (R.M.)

**Keywords:** human glucocerebrosidase, recombinant protein, *Nicotiana benthamiana*, root culture, plant-made pharmaceutical

## Abstract

Gaucher disease is caused by a deficiency of the enzyme glucocerebrosidase (GCase). Currently, enzyme-replacement therapy using recombinant GCase produced in mammalian cells is considered the most effective treatment. Plants are an attractive alternative host for recombinant protein production due to the low cost of large-scale production and lack of risk of contamination by human pathogens. Compared to whole plants, root cultures can grow faster. Therefore, this study aimed to produce recombinant GCase in a *Nicotiana benthamiana* root culture. Root culture of a GCase-producing transgenic plant was induced by indole-3-acetic acid at the concentration of 1 mg/L. Recombinant GCase was successfully produced in roots as a functional protein with an enzyme activity equal to 81.40 ± 17.99 units/mg total protein. Crude proteins were extracted from the roots. Recombinant GCase could be purified by concanavalin A and phenyl 650C chromatography. The productivity of GCase was approximately 1 µg/g of the root. A *N*-glycan analysis of purified GCase was performed using nano LC/MS. The Man_3_XylFucGlcNAc_2_ structure was predominant in purified GCase with two plant-specific glycan residues. This study presents evidence for a new, safe and efficient system of recombinant GCase production that might be applied to other recombinant proteins.

## 1. Introduction

Gaucher disease is an inherited lysosomal storage disorder caused by mutation of the *GBA1* (acid-β-glucosidase) gene, which is located on chromosome 1. The mutation leads to deficiency of a lysosomal enzyme called glucocerebrosidase (acid β-glucosidase or GCase; EC: 4.2.1.25), which catalyzes the hydrolysis of glucosylceramide to glucose and ceramide [1,2]. A lack of GCase leads to accumulation of glucosylceramide in the lysosomes of macrophage cells, resulting in the symptoms of Gaucher disease’s, including bone fractures and pathological enlargement of the spleen and liver [3]. In the general population, the frequency of Gaucher disease is approximately 1/50,000 births [2,4]. Enzyme replacement therapy (ERT) has been used as one of the most efficient treatments for Gaucher disease type I. There are several commercial recombinant GCases for ERT, including imiglucerase (Cerezyme^®^; Genzyme Corporation, Cambridge, MA, USA), velaglucerase alpha (VPRIV^®^; Shire Plc, Dublin, Ireland) and taliglucerase alpha (Elelyso^®^; Pfizer, New York, NY, USA), which are produced in Chinese hamster ovary (CHO) cells, human fibroblasts and carrot cells, respectively. Treating patients with the recombinant enzyme can reduce both spleen and liver sizes as well as improve bone health [3,5]. 

Mammalian cells have several advantages for use in recombinant protein production, including post-translational modification and high expression levels. Currently, around 60% of recombinant pharmaceutical proteins are produced in mammalian cells [6,7,8]. However, such methods present the problems of low scale-up capacity and the risk of contamination by human pathogens. In 2009, Vesivirus 2117 was detected in six bioreactors used for the production of Cerezyme^®^. As a result, Genzyme’s Allston Landing facility in Massachusetts had to shut down, leading to depletion of Cerezyme^®^ supplies for ERT [6,9]. Like mammalian cells, plants also perform post-translational modifications including glycosylation; moreover, they offer many advantages for recombinant protein production, such as low cost for large-scale production and no risk of contamination by human pathogens [6]. There are many researches focusing on recombinant GCase production using plant expression systems such as *Arabidopsis thaliana* complex-glycan-deficient mutant [10], transgenic *Nicotiana benthamiana* [11], and rice suspension cells [12]. *N. benthamiana* is widely used as a host for heterologous expression because it can easily be infected with plant pathogens. This characteristic is useful for foreign gene transformation. *N. benthamiana* has a susceptibility to several viruses many of which could not infect the *A. thaliana*. The number of publications using *N. benthamiana* has been increasing. Therefore, it is easy to exchange knowledge for plant cultivation across laboratories [13,14].

Root expression systems have been increasingly used for recombinant protein production because they can be scaled up easily and allow for continuous large-scale production. Root culture is less sensitive to mechanical damage and it is easy to separate the biomass (root) from the medium. In previous research, the transgene expression in roots has been more stable than the suspension cells [15,16]. Normally, the hairy roots are induced by infection with *Agrobacterium rhizogenes* [16]. Indole-3-acetic acid (IAA) is the most common plant hormone belonging to the auxin class. IAA is involved in plant growth and development [17,18]. In a previous study, IAA could induce the root elongation in maize [19].

*N*-glycosylation plays an important role in pharmaceutical protein production, especially with regard to the bioactivity, solubility and stability of proteins. Currently, there are several pharmaceutical products that exist as glycosylated proteins, including antibodies, enzymes and hormones [20]. Two plant-specific glycan residues (β(1,2)-xylose and core α(1,3)-fucose) would concern the immunogenicity as the detection of antibodies in human sera as an IgE binding carbohydrate determinants. However, those specific antibodies could not react to all glycopeptides containing β(1,2)-xylose and core α(1,3)-fucose [21,22]. The commercial GCase produced in carrot cell suspension (Taliglucerase alfa: Protalix Biotherapeutics, Carmiel, Israel) is the first plant-made pharmaceutical protein that was approved by the United Stated Food and Drug Administration (FDA) for use in humans. The current data from clinical trials showed no significant differences between GCase produced in plants and mammalian cells in all parameters for Gaucher disease treatment and clinical improvement [23].

In this research, we produced recombinant GCase in *N. benthamiana* root cultures induced in a liquid medium. The extracted and purified GCases were characterized by an enzymatic activity assay and *N*-glycan analysis. 

## 2. Results

### 2.1. Root Generation from Leaves of a GCase-Producing N. benthamiana Plant

**Figure 1:**
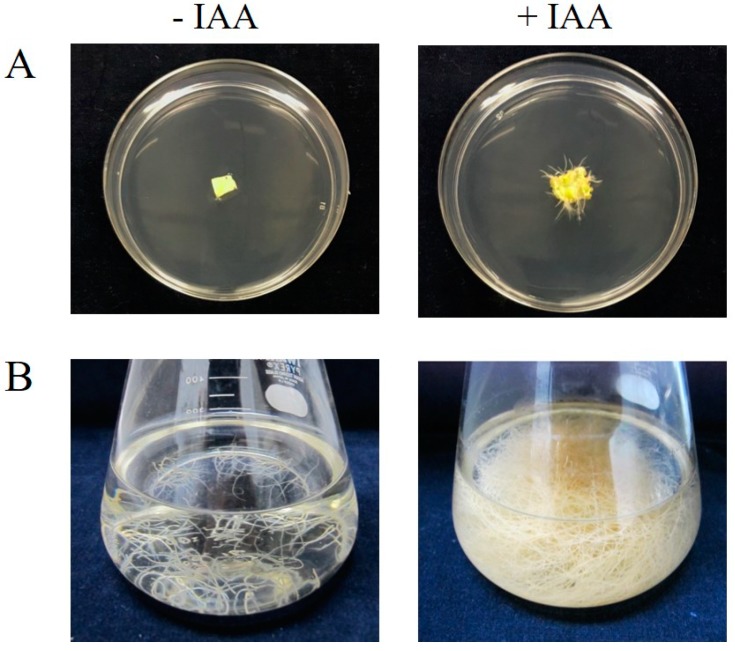
Root induction by IAA on MS solid and liquid media. (**A**) Leaf of transgenic GCase-producing *N. benthamiana* before (left) and after 4 weeks (right) of culture on an MS agar plate supplemented with IAA (1 mg/L). (**B**) Roots of transgenic GC-producing *N. benthamiana* cultured in MS liquid medium supplemented with IAA (1 mg/L) before (left) and after (right) 4 weeks of cultivation. The experiment was performed in triplicate.

Transgenic GCase-producing *N. benthamiana* (At-GC-HSP19) was generated in our previous study [11]. Leaves of the transgenic plant were cut and placed on a Murashige-and-Skoog (MS) medium plate (4.3 g/L MS medium, 30 g/L sucrose and 0.3% gellan gum) and supplemented with IAA (1 mg/L). After 4 weeks of incubation at 25 °C under a 16-h light and 8-h dark condition, roots of transgenic GCase-producing plants could be observed on MS plates supplemented with IAA (Figure 1A). Such roots were not generated on the negative control MS plates without IAA supplementation. In the case of liquid medium, 0.2 g of transgenic GCase-producing roots (9.20 ± 1.84 mg dry root weight) were cut and transferred to MS liquid medium supplemented with IAA (1 mg/L). After cultivation at 25 °C with shaking in a dark condition for 4 weeks, the number of roots was significantly increased as shown in Figure 1B (1456.93 ± 71.10 mg dry root weight). In addition, a small part of the roots could be cut and transferred to other fresh media for new cultivation batches. The rate of root generation in liquid media was higher than that in solid media. However, IAA at a final concentration of 1 mg/L could induce root generation in both solid and liquid media after incubation at 25 °C.

### 2.2. Production of Active GCases in Root Cultures

**Figure 2:**
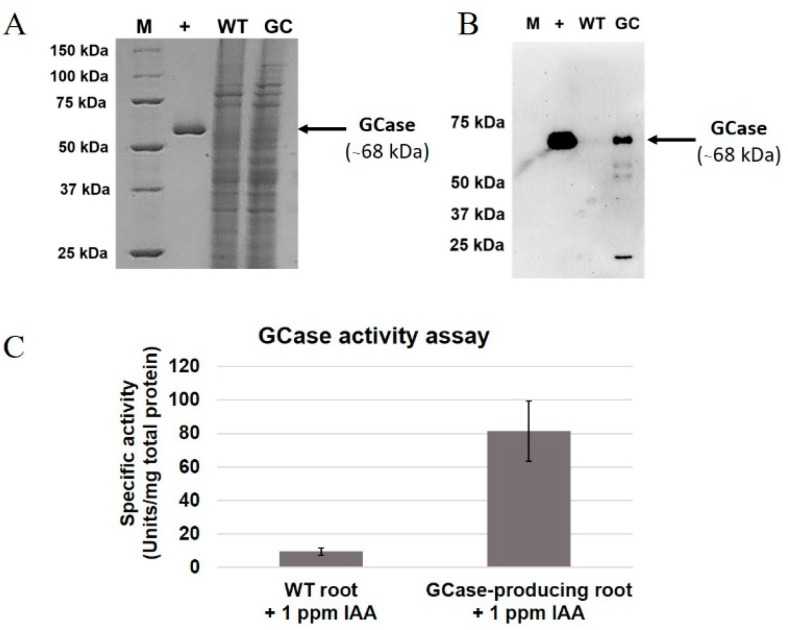
Analysis of GCase produced from root culture. The panels show the results of (**A**) sodium dodecyl sulfate polyacrylamide gel electrophoresis (SDS-PAGE) analysis and (**B**) Western blot analysis and (**C**) the GCase enzymatic activity of total proteins produced from wild-type (WT) and GCase-producing *N. benthamiana* root culture after induction by IAA (1 mg/L). The experiment was performed in three replications. M, precision plus protein standards marker; +, Cerezyme^®^; WT, wild type; GC, GCase. Arrows indicate the GCase band (68 kDa).

Proteins from roots of GCase-producing *N. benthamiana* (0.1 g) were extracted using liquid nitrogen and GCase extraction buffer. A GCase enzymatic activity assay was then performed as described in a previous study [24]. Results of sodium dodecyl sulfate polyacrylamide gel electrophoresis (SDS-PAGE), Western blot and the GCase enzymatic activity assay are shown in Figure 2. The total protein extracted from root cultures was analyzed in comparison to Cerezyme^®^ as a positive control. The total protein extracted from wild-type (WT) *N. benthamiana* was used as a negative control. The putative GCase band was observed at about 68 kDa in the extracts of GCase-producing *N. bentahmiana* but not in the WT as shown in Figure 2A. From the blot results, GCase was detected using anti-GCase antibody (Figure 2B). On the other hand, no GCase band was detected from the *N. benthamiana* WT. The crude protein was subjected to GCase enzymatic activity assay (Figure 2C). GCase-producing roots showed higher enzymatic activity (81.40 ± 17.99 units/mg total protein) compared to wild-type plants (9.60 ± 2.11 units/mg total protein). Therefore, it could be concluded that GCase was successfully produced. The GCase was then extracted from root cultures as an active protein.

### 2.3. Purification of GCase from Root Culture

**Figure 3:**
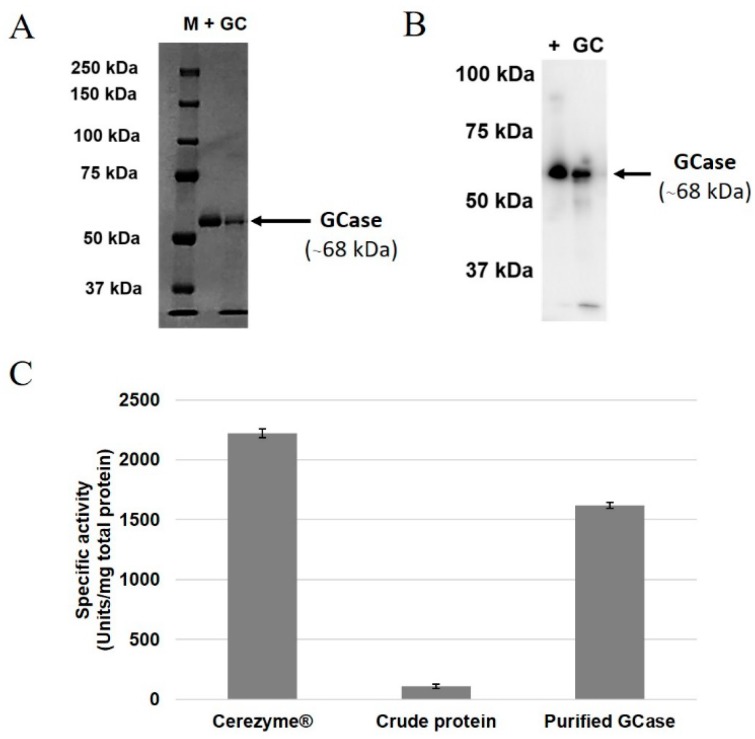
Analysis of purified GCase extracted from root culture. (**A**) SDS-PAGE analysis, (**B**) Western blot analysis, and (**C**) GCase enzymatic activity. Primary antibody, anti-GCase from rabbit; secondary antibody, anti-rabbit-IgG, horseradish peroxidase (HRP)-linked. M, precision plus protein standards marker; +, Cerezyme^®^; WT, wild type; GC, GCase. Arrows indicate the GCase band (68 kDa).

Next, 10 g of the GCase extracted from root cultures was purified. The amount of purified GCase was 1 µg/g of root culture. The purified GCase was detected by SDS-PAGE and Western blot as a single 68-kDa band (Figure 3A,B). The enzymatic activity of the crude protein, purified GCase and Cerezyme^®^ (positive control) are shown in Figure 3C. The enzymatic activity of purified GCase (1618.78 ± 23.57 units/mg total protein) was increased compared to the crude protein (107.91 ± 17.99 units/mg total protein). On the other hand, Cerezyme^®^ showed the highest GCase enzymatic activity (2223.09 ± 36.62 units/mg total protein).

### 2.4. N-Glycan Analysis of Purified GCase

**Figure 4:**
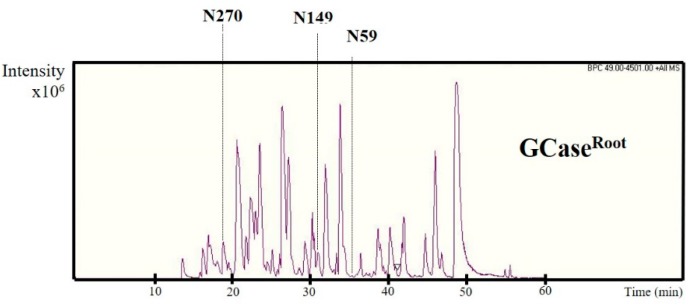
The spectra of glycopeptides derived from purified GCase extracted from root culture. The purified GCase was digested with trypsin and injected to a nano LC-MS/MS. N represents the *N*-glycosylation site at positions 270, 149 and 59.

**Figure 5:**
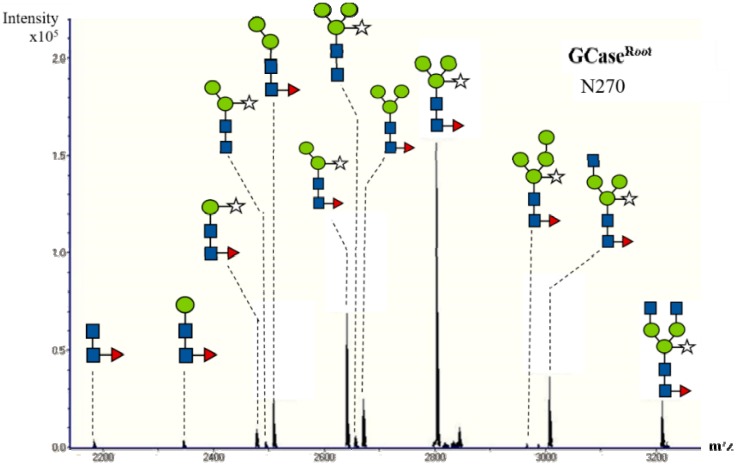

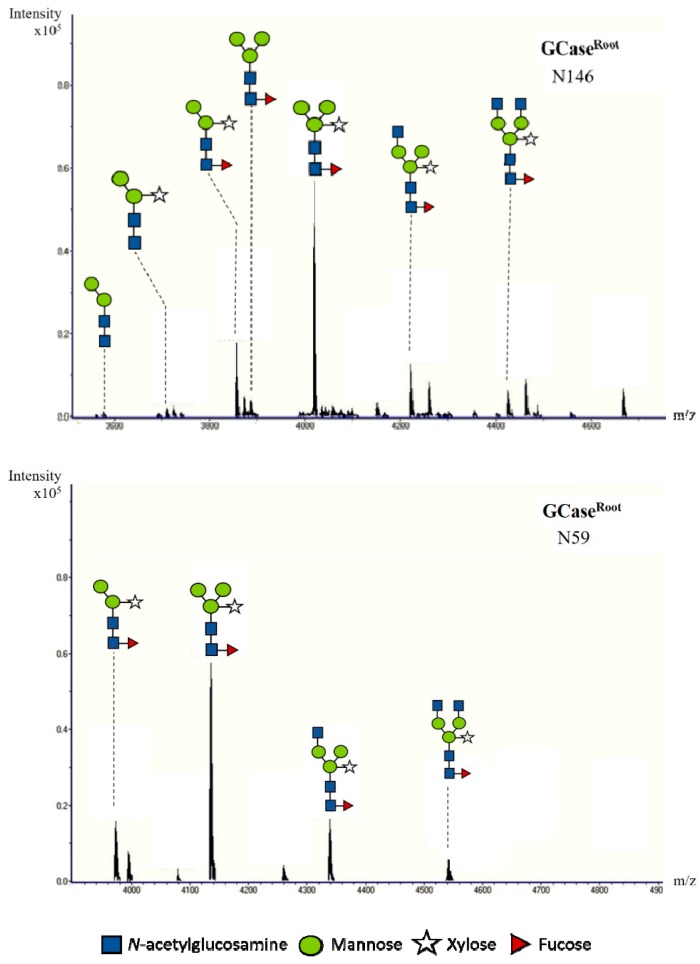
*N*-glycan analysis of purified GCase using nano LC/MS. The glycopeptides with *N*-glycosylation at positions N59, N146 and N270 are shown.

The *N*-glycan analysis of the purified GCase (500 ng) was performed using a nano LC/MS. The purified GCase was digested with trypsin in order to generate glycopeptides. Then the samples were subjected to high-performance liquid chromatography (HPLC) and mass spectrometry, respectively. The elution pattern of trypsin-digested peptides from purified GCase is shown in Figure 4. Previous studies showed the glycan profile at five *N*-glycosylation sites of GCase; only the first four positions, N270, N146, N59 and N19, are usually detected in the wild-type GCase [25]. In this study, glycosylation was found in only three positions (Figure 5): N270, N146 and N59, representing glycosylation at Asparagine positions 270, 146 and 59, respectively. Then, the *N*-glycan structures of each position were analyzed from deconvoluted mass spectrometry (MS/MS) spectra using Compass DataAnalysis version 4.0 (Bruker Daltonics, Bremen, Germany), as shown in Figure 5. The compositions of *N*-glycan chain structures detected from purified GCase are shown in Table 1. The structure Man_3_XylFucGlcNAc_2_ (Man, mannose; Xyl, xylose; Fuc, fucose; GlcNAc, *N*-Acetylglucosamine), which contains both plant-specific glycan residues (β1,2-xylose and α1,3-fucose) was predominant in purified GCase produced from roots at position 270 (43.3%), 146 (56.8%), and 59 (58.7%). The structure Man_2_XylFucGlcNAc_2_ was also detected at positions 270 (19%), 146 (17.7%) and 59 (16.1%). In addition, GlcNAcMan_3_XylFucGlcNAc_2_ was found at all three positions, N270 (10.1%), N146 (12.6%) and N59 (16.7%). 

## 3. Discussion

Recombinant GCase is an important enzyme used in ERT for Gaucher disease type I. Currently, most commercial GCase is produced in mammalian cells (Cerezyme^®^; Genzyme Corporation). However, there are problems with this system, namely its very high cost (USD $200,000/one patient per year) and potential for viral contamination [9,26]. Plant expression systems provide economic benefits for recombinant protein production without the risk of contamination by human virus [15,16]. Therefore, this research was focused on the production of human GCase in *N. benthamiana* using a root-specific expression system. 

The transgenic GCase-producing *N. benthamiana* (At-GC-HSP19) used in this study was generated as described in our previous research [11]. The roots of the transgenic plant could be induced in both solid and liquid media supplemented with IAA at the concentration of 1 mg/L (Figure 1). For GCase analysis, crude proteins were extracted from roots and analyzed by SDS-PAGE, Western blot and GCase enzymatic assay. The GCase enzymatic assay detects β-glucosidase activity, which can hydrolyze the β-glycosidic linkage at non-reducing residues of β-glucosides and oligosaccharides [27]. Crude protein extracted from *N. benthamiana* WT showed a small amount of β-glucosidase enzymatic activity (9.60 ± 2.11 units/mg total protein), which was considered to originate from internal β-glucosidase [28,29], since the β-glucosidase found in plants plays important roles in lignification, chemical defense and phytohormone regulation. In previous research, recombinant crude GCase was produced from leaves of transgenic plants with GCase enzymatic activity of approximately 44.5 units/mg total protein [11]. The crude GCase produced from root cultures in our study provided higher enzymatic activity (81.40 ± 17.99 units/mg total protein). Therefore, this root system might be useful for GCase production. 

The produced GCase was successfully purified using both Con A affinity chromatography and Phenyl 650M hydrophobic chromatography (Figure 3). The GCase band (68 kDa) was detected in both SDS-PAGE and Western blot analysis. The amount of purified GCase in this study was about 1 µg/g of root. The root expression system provides advantages in recombinant protein production, especially using scale-up and a continuous-production process [15,16]. In the case of whole plants, it might take 2 months before the seed becomes a mature plant. After the leaves have been cut and used for protein extraction, the next batch of production has to be generated from seeds again [11] or re-generated from leaf-cut plants. On the other hand, a small amount of root culture can be transferred to new medium before protein extraction is performed; this root culture requires only 2–4 weeks for growth. For these reasons, root specific-expression systems are more efficient in term of reproductivity. In the purification process, the elution buffer for Con A chromatography used in this study was different from that used in the previous research. This study used d-glucose, which binds to Con A resin with weaker affinity than the α-methyl-d-mannoside used in the previous study [11,30]. However, glucose was chosen because of the lower price compared to α-methyl-d-mannoside, thereby reducing the cost of the production process.

*N*-glycosylation in plants is important because it is related to stability, protein conformation, and biological activity [31]. In this study, *N*-glycan was analyzed by a nano LC/MS. The HPLC elution pattern and *N*-glycan structure of purified GCase from roots are shown in Figure 4 and Figure 5, respectively. The compositions of *N*-glycan residues in the purified GCase extracted from root culture and purified GCase extracted from leaves of a transgenic plant [24] are shown in Table 1. Four glycan residues were detected in the previous research (N270, N146, N59 and N19). However, only three *N*-glycan residues were found in this study (N270, N146 and N59). The N19 glycan could not be detected, for reasons unknown to us. The structure Man_3_XylFucGlcNAc_2_, which contains plant-specific β1,2-xylose and α1,3-fucose, was predominant in both the purified GCase from root cultures and that from leaves at all *N*-glycan residues. On the other hand, ManFucGlcNAc_2_, Man_4_XylFucGlcNAc_2_, ManXylFucGlcNAc_2_ and Man_2_GlcNAc_2_ were found only in purified GCase produced from root cultures, with a low percentage at different *N*-glycan residues (1%, 0.4%, 2.6% and 1%, respectively). The other compositions of *N*-glycan structure did not show much difference between the purified GCases produced in the roots and leaves of transgenic plants. Therefore, it could be concluded that the recombinant GCase produced in roots was not different from the GCase produced in leaves in terms of *N*-glycan structure. The differences between *N*-glycan from mammalian and plant cells are plant-specific residues, β(1,2)-xylose and α(1,3)-fucose. These two residues might induce the immunogenicity as detection of specific antibodies in mammals and humans. However, the antibody specific to those two structures did not react to all of glycoproteins with β(1,2)-xylose and α(1,3)-fucose residues as analyzed in a further study. In addition, the first plant-made pharmaceutical protein approved by FDA (Taliglucerase alfa) contains about 90% of plant-specific glycan structures and no side effect of these structures have been reported in Phase III clinical trials [21,22,32]. Therefore, the purified GCase produced in the root system should be safe for the treatment of Gaucher disease.

## 4. Materials and Methods

### 4.1. Transgenic GCase-Producing Plants and Growth Condition

Transgenic GCase-producing *N. benthamiana* (At-GC-HSP19) was generated as described in a previous study [11]. The seeds of the transgenic plant were sterilized by PPM^TM^ solution (Plant Preservative Mixture; Plant Cell Technology, Washington, DC, USA) and germinated after incubation on Murashige-and-Skoog (MS) medium plates supplemented with bialaphos (10 μg/mL) at 25 °C for 2 weeks under a 16-h light and 8-h dark condition. Each plant was transferred to a sterile MS-medium pot (100 mL/pot) and incubated at 25 °C.

### 4.2. Root Generation from Leaves and Roots of Transgenic Plants

Each leaf and root of the transgenic GCase-producing *N. benthamiana* was cut and placed on two different MS medium plates, with and without the addition of 1 mg/L IAA. Both plates were supplemented with bialaphos (10 mg/mL). Plates were incubated at 25 °C under 16-h light and 8-h dark conditions for 4 weeks. The experiment was performed in three replications.

### 4.3. Root Culture Induction by Indole-3-Acetic Acid (IAA) in Liquid Media

Roots of the transgenic GCase-producing *N. benthamiana* were cultured at 25 °C in 100 mL of MS liquid medium supplemented with IAA at the concentration of 1 mg/L and bialaphos (10 μg/mL) at 33 g. The MS media was changed and replaced with fresh media every 2 weeks. The experiment was performed in triplicate. 

### 4.4. Protein Extraction from Root Culture

Protein extraction was adapted from a previous study [24]. Briefly, roots were homogenized by grinding with a mortar and pestle under liquid nitrogen. Root powder was suspended in GCase extraction buffer (20 mM Tris-HCl pH 7.0, 150 mM NaCl, 0.5% taurocholic acid and 1 mM phenylmethylsulfonyl fluoride) and centrifuged at 12,000× *g* at 4 °C for 10 min. The supernatant was collected and analyzed by GCase enzymatic assay, Bradford assay, SDS-PAGE and Western blot [24].

### 4.5. GCase Enzymatic Assay

The enzymatic activity of GCase was determined as described previously [10]. The extracted protein was co-incubated with GCase activity assay buffer (60 mM phosphate-citrate buffer pH 6.0, 4 mM β-mercaptoethanol, 1.3 mM EDTA, 0.15% Triton X-100, 0.125% taurocholic acid and protease inhibitor) and 4-methylumbelliferyl β-d-glucopyranoside (4-MUGP; Wako, Osaka, Japan) at 37 °C for 1 h. The reaction was stopped by glycine buffer (0.2 M glycine, 0.125 M sodium carbonate, pH 10.7). The fluorescence of the reaction was detected using a F-25000 fluorescence spectrophotometer at λ_ex_ = 365 nm and λ_em_ = 460 nm (Hitachi, Tokyo, Japan). GCase activity was calculated in comparison to the standard curve of 4-methylumbelliferone (4-MU; Wako), which is a reaction product. One unit of GCase enzyme was defines as the amount of enzyme required to release 1 nmol of 4-MU/min. The specific activity was calculated as unit/mg of total extracted protein. 

### 4.6. Western Blot Analysis

The protein extracted from roots after 4-week cultivation was separated by polyacrylamide gel electrophoresis (10% gel) and transferred to a polyvinylidene difluoride membrane (Millipore, MA, USA). The membrane was incubated with 5% skim milk in phosphate buffered saline with Tween 20 (PBST) solution (1.47 mM KH_2_PO_4_, 10 mM Na_2_HPO_4_, 2.7 mM KCl, 137 mM NaCl, 0.05% Tween-20, pH 7.4) for 30 min, followed by incubation with the polyclonal anti-glucocerebrosidase rabbit antibody (Sigma-Aldrich, St. Louis, MO, USA) for 1 h. The antibody was diluted at 1:5000 in PBST. After being washed with PBST, the membrane was incubated with anti-rabbit IgG (Sigma-Aldrich, St. Louis, MO, USA) diluted 1:5000 in PBST for 1 h. The protein was detected after incubation with Luminata^TM^ (Millipore, Darmstadt, Germany) at room temperature for 5 min using a FluorChem FC2 Imaging System (American Laboratory Trading, East Lyme, CT, USA).

### 4.7. GCase Purification from Root Culture

The purification protocol was developed from the previous study [20]. Roots of transgenic plants (10 g) were homogenized by grinding with a mortar and pestle in liquid nitrogen. The root powder was resuspended in GCase extraction buffer (80 mL) and incubated on ice for 20 min before centrifugation (10,000× *g* for 20 min). The supernatant was separated using a filter membrane and loaded into a Con A-Agarose column (3 cm × 18 cm) (J-Oil Mills, Tokyo, Japan) using a peristaltic pump (Perista Pump SJ-1211; ATTO, Tokyo, Japan) as a recycling system with an intensity of 10 at 4 °C for 24 h. The column was washed using Con A buffer (500 mM NaCl, 1 mM MgCl_2_, 1 mM MnCl_2_, 1 mM CaCl_2_ in 20 mM Tris-HCl pH 7.0). The protein was eluted with 0.5 M glucose in a GCase assay buffer. The eluted proteins were analyzed by GCase enzymatic assay. The active fractions were collected and resuspended in 2 M NaCl. The supernatant was loaded on a phenyl-650C column (1.5 cm × 20 cm) (Tosoh, Tokyo, Japan). The protein was eluted by decreasing the concentration of NaCl. The eluted protein was analyzed using a GCase enzymatic assay. The active fraction was collected and concentrated using a Vivaspin column with 30-kDa membrane (GE Healthcare UK, Buckinghamshire, UK). The concentrated protein was analyzed by Bradford assay, SDS-PAGE and Western blot.

### 4.8. N-Glycan Analysis

The glycan analysis was performed as described in a previous study [24]. The purified GCase from root culture was separated using SDS-PAGE and stained by Coomassie brilliant blue (CBB) solution. Reduction and alkylation of the GCase band excised from the gel were performed as described previously [33]. Proteins were digested by Trypsin Gold (Promega, WI, USA) at 50 °C for 1 h. Peptides were extracted from the gel using 1% trifluoroacetic acid in 60% acetonitrile, followed by evaporation at 30 °C for 2 h. Peptides were dissolved in 0.1% formic acid and applied to an ESI-Qq-TOF mass spectrometer (microTOF-Q II; Bruker Daltonics, Billerica, MA, USA) for *N*-glycan analysis. The micrOTOF control software (Version 4.0, Bruker Daltonics, Bremen, Germany) was used to control the complete system (Bruker Daltonics). Compass DataAnalysis version 4.0 was used for glycan analysis. 

## 5. Conclusions

In this study, we present an alternative plant host system for the production of recombinant GCase in root culture. Root culture of transgenic GCase-producing *N. benthamiana* was induced by IAA in both solid and liquid media. GCase was then produced and purified from the root culture as a functional protein with 1618.78 ± 23.57 units/mg total protein. The productivity of GCase was approximately 1 µg/g of root. Compared to GCase produced in leaves of whole plants, our system showed lower productivity. However, this system provided better benefits in terms of shorter cultivation time and continuous production process. The *N*-glycan analysis of purified GCase was also performed, and the results were not very different to those of the purified GCase produced in leaves from our previous study. This system could be applied to culture the transgenic glycoengineered *N. benthamiana* plant (GC*^gnt1^*) for the production of GCase with less plant-specific glycans. In addition, the scale-up and optimization of the purification process should be studied in the future. 

## Figures and Tables

**Table 1:** Composition of the *N*-glycan structure attached to purified GCase from leaves [24] and purified GCase from root culture.

Structure	Amount of Composition (%)					
	N270		N146		N59	
	GC^WT^	GC^Root^	GC^WT^	GC^Root^	GC^WT^	GC^Root^
FucGlcNAc_2_	-	1.1	-	-	-	-
ManFucGlcNAc_2_	-	1.0	-	-	-	-
Man_2_FucGlcNAc_2_	6.7	6.6	-	-	-	-
Man_3_FucGlcNAc_2_	9.2	6.9	4.6	3.7	4.0	-
Man_2_GlcNAc_2_	-	-	-	1.0	-	-
GlcNAc_2_Man_3_XylFucGlcNAc_2_	5.2	6.6	-	6.2	7.2	8.5
GlcNAcMan_3_XylFucGlcNAc_2_	10.0	10.1	5.2	12.6	19.5	16.7
Man_4_XylFucGlcNAc_2_	-	0.4	-	-	-	-
Man_3_XylFucGlcNAc_2_	43.5	43.3	65.5	56.8	56.1	58.7
Man_3_XylGlcNAc_2_	1.5	1.6	5.5	-	1.8	-
Man_2_XylFucGlcNAc_2_	23.9	19	16.5	17.7	11.3	16.1
Man_2_XylGlcNAc_2_	-	0.8	2.7	2.0	-	-
ManXylFucGlcNAc_2_	-	2.6	-	-	-	-
	**100**	**100**	**100**	**100**	**100**	**100**

## Abbreviations

IAA	Indole-3 acetic acid
Man	Mannose
GlcNAc	*N*-Acetylglucosamine
Xyl	Xylose
Fuc	Fucose
4-MUGP	4-Methylumbelliferyl β-d-glucopyranoside
4-MU	4-Methylumbelliferone
